# Development of a workflow to analyze autoinflammatory-associated genes using AccessArray™ system and next generation sequencing

**DOI:** 10.1186/1546-0096-13-S1-O81

**Published:** 2015-09-28

**Authors:** E Gonzalez-Roca, E Ruiz-Ortiz, MC Anton, S Plaza, A Mensa-Vilaro, J Yagüe, JI Arostegui

**Affiliations:** 1 Hospital Clinic, Immunology, Barcelona, Spain

## Introduction

Monogenic autoinflammatory diseases are a group of genetic conditions characterized by a dysregulation of inflammatory response that include more than 20 diseases. Despite the fact that they are different clinical entities, several signs and symptoms are shared, making their differential diagnosis difficult. Moreover, in most cases, a definitive diagnosis is exclusively achieved by a positive genetic study. The need to analyze different genes makes this process costly in terms of both time and money. The arrival of next generation sequencing into clinics can overcome these problems.

## Objective

To develop a complete workflow to analyze simultaneously the most common autoinflammatory-associated genes.

## Materials and methods

A panel of 80 different primer pairs was designed according to AccessArray™ and PGM Ion Torrent platform specifications. This panel covers *MEFV* (all exons except 2), *TNFRSF1A* (exons 2-to-7), *MVK* (all exons), *NLRP3* (all exons), *NOD2* (exons 4, 8, 11) and *PSTPIP1* (exons 10-11) genes. Libraries were prepared using 48.48 AccessArray™ chips and sequenced in the PGM platform using 400 bp kits. Quality control, mapping and variant calling were performed using Torrent Server and variant annotation using Ion Reporter Suite. Coverage information was analyzed with BEDtools software.

A set of 25 control samples was used to validate our sequencing and data analysis workflow. Then, a total of 288 patient samples were analyzed. Any possible disease-causing mutation detected was confirmed by Sanger sequencing.

## Results

The results obtained in control samples were concordant with the expected known genotypes. All germline mutations (100%) and 4 out of 5 somatic mutations (80%) were detected. Only a sample harboring extremely-low somatic *NLRP3* mosaicism (less than 4%) was not initially called by our pipeline. In the analysis of 288 samples, all mutations detected were confirmed by Sanger sequencing. Moreover, in one of the analyzed patients, a somatic *NLRP3* mutation (12%) was identified in the routine screening.

## Conclusion

We have designed a complete workflow to simultaneously analyze the most common autoinflammatory-associated genes in a clinical setting. This workflow allowed us to identify all of the germline mutations and most of the somatic mutations previously detected in our control group. All the detected mutations in new samples where confirmed by Sanger sequencing. This NGS-based approach also enabled us to detect a novel case of somatic *NLRP3* mosaicism in a patient with disease symptoms compatible with CAPS.

